# Silos and rigid processes: Barriers to the successful implementation of the Older prisoner Health and Social Care Assessment and Plan

**DOI:** 10.1177/00258024221141641

**Published:** 2022-11-29

**Authors:** K. Forsyth, G. Daker-White, L. Archer-Power, J. Senior, D. Edge, R.T. Webb, J. Shaw

**Affiliations:** 1 Health and Justice Research Network, 5292 The University of Manchester, Manchester, UK; 2 Population Health, 5292 The University of Manchester, Manchester, UK; 3 Lancashire Police, Lancashire, UK; 4 Equality, Diversity and Inclusion Research Unit, 5292 The University of Manchester, Manchester, UK; 5 School of Health Sciences, 5292 The University of Manchester, Manchester, UK

**Keywords:** Older adults, prison, care plans, assessment, health services, social care

## Abstract

Older adults are the fastest growing sub-group in prisons. They have complex health, social care and custodial needs and often the support they receive is sub-optimal. The Older prisoner Health and Social Care Assessment and Plan (OHSCAP) aimed to better meet these inter-related needs. As part of a wider study, a randomised controlled trial was conducted to evaluate the OHSCAPs effectiveness in meeting older prisoners’ health, social care and custodial needs in comparison to treatment as usual. This article describes the nested qualitative study which aimed to explore the barriers and facilitators to the effective implementation of the OHSCAP. Semi-structured interviews were conducted with older adults (n = 14) and staff members t (n = 12). Data was analysed using the framework method. Three overarching key themes were identified. These were: (1) balancing care and custodial requirements; (2) prison, health and social care silos; and (3) rigid prison processes. Prison is an important opportunity to engage residents and improve public health. Cultural and strategic change is required for health, social care and custodial interventions, such as the OHSCAP, to be successfully implemented into prison settings.

## Introduction

As many as 10.74 million individuals are incarcerated worldwide.^1^ The USA has the highest incarceration rate in the developed world (655 per 100,000 population). Individuals in prison are a marginalised and socially excluded group who experience poorer health than their counterparts residing in the wider community.^2,3^ Consequently, the health of prisoners presents a significant public health challenge and prison is an opportunity to engage this socially disadvantaged group.^4^

There has been considerable increase in the number of older prisoners across many developed countries, including the USA,^5^ Japan,^6^ Canada,^7^ Australia,^8^ France^9^ and England and Wales.^10^ Sixteen percent of the prison population in the USA are aged 55 and over,^11^ and 17% of the prison population in England and Wales are now aged 50 and over (13,283). This includes 5069 individuals aged 60 plus; triple the number there were 15 years ago.^12^

Older adults residing in prison have multiple health problems. They have more complex health needs than their peers in the community and younger individuals in prison.^11,13^ In England and Wales, it is estimated that between 85% and 93% have some form of physical illness.^14,15^ Older prisoners often also have complex social care needs that prisons are ill-equipped to manage.^16^ In the USA, one-fifth of older prisoners struggle with activities of daily living.^11^ Older adults residing in prison experience intense anxieties about release and they typically perceive their release planning to be non-existent.^17^

Providing healthcare in prisons is a universal challenge internationally for four key reasons: (1) the conflict between care provision and security; (2) problems in achieving equivalent care to that received by all persons in general population; (3) difficulties ensuring timely access to services overcoming security restrictions; and (4) achieving clinical independence from custodial management arrangements.^4^ Globally, there is insufficient evidence about how prison healthcare should be structured or funded.^4^ The World Health Organisation (WHO) stated that the responsibility for health should lie with health authorities rather than criminal justice organisations.^4^ In the USA, with private healthcare provision there are multiple different models of prison healthcare in comparison to the UK, where the National Health Service (NHS) has been responsible for healthcare in prison since 2006.^18^ The introduction of the Care Act (2014) in England and Wales clarified that the responsibility for the social care of prisoners lies with the local authority/council where the individual is incarcerated. There is a dearth of literature concerning social care provision within prisons in the USA.

In England and Wales, there is no national strategy for older prisoners in spite of repeated calls for one to be developed. Consequently services are sub-optimal and *ad hoc*.^19^ In response to this, the OHSCAP was developed and implemented by an Action Learning Group (including prisoners, NHS staff and prison staff) as part of a previous study.^19^ The OHSCAP is a structured approach for better identifying and managing the health and social care needs of older prisoners. The OHSCAP is paper-based and information collected is uploaded onto prison computer programmes. It consists of an assessment, a care plan and reviews of these, with the assessment conducted approximately 1 to 2 weeks after an older adult enters prison.

The data that are reported in this article were collected as part of a larger study^20^ which aimed to evaluate the effectiveness and acceptability of the OHSCAP in a randomised controlled trial (RCT). The objectives of the larger study were to: (1) evaluate the efficacy of the OHSCAP in meeting older male prisoners’ health and social care needs,^21^ (2) assess its cost-effectiveness, (3) assess the quality of care plans produced^22^ and (4) explore the experiences of older prisoners and staff. The qualitative aspects of the study (objective 4) are reported in this article. This article explores the challenges of implementing health and social care initiatives within prisons more broadly.

## Methods

The core study consisted of a parallel two group RCT with 1:1 individual participant allocation to either the OHSCAP intervention plus treatment as usual (TAU) (intervention group) or TAU alone (control group). TAU included the standard, non-age-specific health assessment carried out at prison entry.^23^ Support provided as TAU varied from prison to prison, but included interventions such as older prisoner social groups, peer carers and ‘healthy man’ checks. Ongoing assessments and interventions followed local procedures at each establishment. Previous research has indicated that the identification of health and social needs and subsequent care planning is generally *ad hoc* and inadequate.^24^ The core study recruited older male prisoners aged 50 and over from a representative sample of 10 prisons in England.

Ethics approval for the study was granted by the Research Ethics Committee for Wales in May 2013 (reference number 13/WA/0108). National Offender Management Service research approval was provided in July 2013 (reference number 2013-115).

Semi-structured interviews were held with staff delivering the intervention. A purposive sampling approach was adopted ensuring prison and healthcare staff were included. Prisoners from all of the 10 sites were also interviewed. The sample was purposive with the intention of including prisoners across a range of ages and varying health and social care needs.

The interview guide format followed the OHSCAP process itself (identifying older adults, health, social and wellbeing issues, care planning and reviews) to ensure that all relevant issues were covered. Prisoners were interviewed between one and four times (mode = 2). They were interviewed as soon as possible after they entered the prison, then immediately after they had received the initial OHSCAP and, if they remained in prison, we went back to interview them after 2 to 3 months. All interviews with staff were audio recorded and lasted for approximately 1 h. It was not possible to audio record interviews with 6 of the 14 prisoners as a result of security restrictions. In these instances, 2 researchers attended the interviews and notes were taken contemporaneously.

All interviews were analysed thematically using a framework method.^25^ In the analysis, data is sifted, charted and sorted in accordance with key issues and themes using five steps: familiarisation; identifying a thematic framework; indexing; charting; and mapping and interpretation.^26^ The framework method produced a matrix of summarised data, which provided a structure to analyse and reduce the data. It also allowed systematic constant comparisons across participants’ transcripts cases to refine themes. It is frequently used in applied research studies where the research questions are predefined.^26^

## Results

Semi-structured interviews were conducted with staff delivering the OHSCAP, including prison officers (*n*  =  5) and healthcare staff (*n*  =  7). All five prison officers had specific roles for supporting vulnerable prisoners (e.g. disability liaison officers). The healthcare workers interviewed included healthcare assistants (*n*  =  3), general nurses (*n*  =  2) and a mental health nurse (*n*  =  1). Additionally, semi-structured interviews were held with 14 prisoners who had received the OHSCAP. None of the individuals approached refused participation and interviews continued until data saturation was reached. The participating prisoners ranged in the age from 50 to 69 years (mean  =  58 years). The most common index/main offence types were sexual (*n*  =  5), and drugs (*n*  =  5). The majority had been imprisoned between one and eight times previously (*n*  =  11). Two of the prisoners had not been incarcerated previously and one had been imprisoned on more than 10 previous occasions.

Three overarching key themes were identified namely: (1) balancing care and custodial requirements; (2) prison, health and social care silos and (3) rigid prison processes.

### Balancing care versus custodial requirements

Participants, particularly staff members, described how prison environments are in essence an interface between two worlds; care and custody (Table 1). They described how this created challenges for supporting older prisoners generally and, specifically, in being able to successfully adhere to the OHSCAP process. This was evident across the two sub-themes of ‘being dismissive of social care needs’; and staff knowledge and understanding.

**Table 1.** Theme one: care versus custody.

Dismissive of social care needs	
1. Prison Officer 6	*I just consider working on the wings like the actual bread and butter of the job,* *…* *But it's not issues like ‘can I have a new flask because I can’t open mine properly’, or; or, ‘can I have a sock aid to help me get my socks on because I can’t bend over properly’; it's proper issues, like ‘I need to ring my mum because she's not well’ and ‘I’ve got no money on my PIN [a person's individual payphone accounts], can you sort this for me’; ‘I can’t get this person cleared for a visit, can you help me’.*
2. Prison Officer 6	*Helping with these things [social care needs] is too care beary.*
Staff knowledge and understanding	
3. Prisoner 3	*I have ups and downs. They don’t understand that I can have good days and bad. I finally got to the library once (by myself`) and it's caused me grief ever since. I’m being penalised for going to the library ‘cause they now expect me to be able to walk everywhere*.
4. Prisoner 7	*I have to stand up for an hour or so because the seat isn’t anywhere near the wall to lean back on. I’ve told them before that I need a seat to sit on, but they won’t give me one.*

#### Dismissive of social care needs

Older adults discussed how prison staff were reluctant to support them with their social care needs. Prison officers often did not perceive that meeting of social care needs was a valuable part of their job. They had not been motivated to be prison officers in order to take on a caring role (Table 1 quote 1). This was even evident for prison officers who were specifically employed to support vulnerable prisoners, such as disability liaison officers. Maintaining a dismissive attitude towards social care issues was part of a culture that helped officers cope with the stresses and strains of the challenging prison environment (Table 1 quote 2). Prison officers reported that they did not have the capacity to assist everyone with all of their problems and helping prisoners with very personal issues could prevent officers maintaining an emotional distance. Maintaining an emotional distance was necessary for them to cope with the highly emotive and challenging work pressures. Healthcare personnel who facilitated the OHSCAP were less dismissive of providing support with activities of daily living. However, they frequently did not perceive social care issues such as managing accommodation housing or finances to be their responsibility.

#### Staff knowledge and understanding

Older adults described prison staff members’ lack of understanding and empathy towards their health and social care needs. One older adult described how prison officers failed to consider the varying nature of his illness and the consequent daily changes to his activities of daily living needs (Table 1, quote 3). Staff often assumed that the older adults were being misleading about their social care needs in a bid to gain attention or privileges. Prisoners indicated that some officers perceived that prisoners were incarcerated primarily as a punishment, and they therefore felt that they should not expect luxuries. They went on to state that these individuals failed to consider the pain and suffering that some prisoners undoubtedly experience when they, for example, have mobility difficulties (Table 1, quote 4).

### Prison, health and social care silos

Staff members considered prison problems and healthcare issues to be distinct and separate (Table 2). Furthermore, prison staff and healthcare personnel did not generally appear to work collaboratively at a strategic level to support prisoners. This was evident in their approach to information sharing and signposting.

**Table 2.** Theme two: health, social and custodial silos.

Information sharing	
1. Healthcare worker 4	*And one [prisoner].* *.* *. he’d lived in children's homes all his life and he’d had a girlfriend that had died, and he had nothing to support him on the outside, and he really didn’t want to come out of prison. So, I really didn’t know what to do about that. I didn’t have enough experience with the prison to know about that, and unless it's a safety issue we’re not allowed to share that sort of information with the prison side of it.*
2. Healthcare worker 3	*Yes, I think there is a prison computer downstairs in admin, but I haven’t used the prison computer for… years and years now. They’re not the simplest either.*
3. Prisoner 4	*It's better that I’m not walking up and down stairs all the time now. It doesn’t matter who it is as* long as I can speak to someone when I need to. I’ve nothing to hide about my health, have I?
Signposting	
4. Healthcare worker 3	*I don’t know if that's [prisoners’ personal finance] really our responsibility or not, or we could do much about it, obviously.., but then if there's nothing we can do about it, there's no point asking the question and building their hopes up, is there?*

#### Information sharing

There was confusion over what information could be shared between healthcare staff and prison officers including in a prisoner's best interests. Older adults actually wanted information about their health to be shared to overcome barriers to receiving appropriate services and accessing facilities. A culture of fear about not adhering to data protection rules predominated over the need to support older adults (Table 2, quote 1). Effective information sharing was further impeded by limited shared access to prison IT systems and poor IT literacy skills. Effective and appropriate information sharing practices are a prerequisite to the successful implementation of the OHSCAP, as well as appropriate care in general.

#### Signposting

There was a general lack of awareness among all staff regarding what services were available to older adults in prison. Staff were largely unaware of how individuals could obtain support, particularly with regards to social care issues such as finance and housing. Therefore, signposting or referring to other services was rare (Table 2, quote 4). The OHSCAP facilitators were not in the position to support older adults if they were not fully aware of the services available to them.

### Rigid prison processes

Throughout the interviews, both prison and healthcare staff described the rigid prison processes that they had to adhere to (Table 3). They frequently followed those procedures, even when completing the OHSCAP processes. Identified sub-themes included process focussed solutions, going ‘above and beyond’, and a lack of accountability.

**Table 3.** Theme three: rigid prison processes.

Process focused solutions	
1. Prisoner 4	*They [staff members] come out with pathways this and pathways that but I don’t think any of them could find a path up their own garden.*
2. Prisoner 9	*Oh yeah, ‘put an app in. See you in a week’. No one's really interested.*
3. Healthcare worker 4 and interviewer Healthcare worker 3 Interviewer Healthcare worker 3 Healthcare worker 4	*Yeah, some of the lads [prisoners] obviously didn’t have their medication when they first came in, but that's because of processes that we’ve got, it can take up to 2 weeks*. They’re quite upset about it. But obviously it was sorted in the end. But I agree with them, why should they have to go without their medication basically. It's been an ongoing issue for a long time. *Right, and is there anything you can do to speed things up?* *It's just the process*. *I mean the process is the process.*
Above and beyond	
4. Prisoner 9	*I just had to speak to [wing staff] and do all of this PINs and stuff and say can we try and rush this number through for him because he's not spoke to anybody.*
5. Prison Officer 6	*We just know that we…because some of them [sighs]…The one…that went to outside hospital and he's come back in and he's on permanent oxygen, you can't do anything in his cell. So, I just took him some crosswords and puzzles, or something to do, because he couldn’t…they wouldn’t take him into association because he can't take his oxygen, and everything else, with him.*
Accountability	
6. Healthcare worker 5	*There is a wheelchair based in the centre, but the brake broke so we could not use it. You ask people to look into it and no one gets back to you.*
7. Prisoner 1	*Well I spoke to them [healthcare staff], and they said, if the tablets don't arrive at the prison, it's not their problem. So…* *whose problem it is?*
*8. Healthcare worker*	*I can I manage the workload…so it slotted into my role. I reduced my caseload in other areas.*

#### Process focussed solutions

Older adults were often frustrated by staff members’ tendency to focus on the process rather than genuinely meeting their needs (Table 3, quote 1). They were particularly exasperated by the applications process, frequently referred to as ‘apps’. This is the written process by which prisoners could access help or support for a wide variety of issues while incarcerated. Prisoners detailed how officers often simply told them to complete an application for every aspect of support that they required, without appearing to pay heed to their specific needs, even if such an application had previously been made and the proposed solution had failed to meet the prisoner's needs (Table 3, quote 2). This could be a coping mechanism for the sheer number of queries that they received and a means of remaining emotionally detached from the prisoners..

#### ‘Above and beyond’

When both healthcare and prison staff felt able and were willing to go ‘above and beyond’ the rigid prison process by being more flexible, it was greatly appreciated by prisoners. This more flexible approach was also beneficial in that it led to improved health and wellbeing outcomes among older prisoners.

It was often the case that issues that became apparent during the OHSCAP assessment were simply being dealt with within the failing rigid prison processes. For example, older adults may have been waiting for their friends’/family members’ telephone numbers to be approved so they could call them from prison. This process is necessary to prevent prisoners contacting victims or other vulnerable individuals. However, sometimes this process can take a number of weeks causing great distress to those who are incarcerated and their family/friends. If the member of staff simply told the prisoner of the process to repeat to get their telephone numbers approved, this had no impact. However, if the staff member was able to investigate (Table 3, quote 4) the cause of the delay and take appropriate actions, stress levels were markedly reduced.

#### Lack of accountability

Both staff and prisoners stipulated that there was a general lack of accountability for resolving prisoners’ health and social care needs across the studied prisons. Examples were provided for how this impacted the effectiveness of the OHSCAP. For example, one healthcare worker described the difficulties that they experienced in allocating wheelchairs to older adults due to no one being accountable for maintaining them (Table 3, quote 6). An older adult also provided the example below of how no one appeared to be accountable for ensuring that medication was received on time (Table 3, quote 7).

When facilitators of the OHSCAP considered recipients to be on their caseload, they were more likely to address their health and social care needs and follow-up actions required (Table 3, quote 8). The OHSCAP process is reliant on facilitators being accountable for ensuring issues raised are addressed.

## Discussion

### Main findings

A RCT found that the OHSCAP did not improve the meeting of older adults’ health and social care needs in comparison to TAU. The audit of OHSCAP assessments found that the intervention was fundamentally not delivered as planned.^27^ The qualitative interviews described in this article explored why this was the case. Three key themes were identified: namely the challenge of balancing care versus custody needs; the existence of prison, health and social care silos; and rigid prison processes.

### Comparisons with the existing literature

The dichotomy of ‘meeting health and social care needs’ versus ‘incarceration’, has resulted in challenges for the effective implementation of health or social care initiatives within prison.^28–30^ Wilmott^28^ identified a need for research to establish how to promote a positive interface and more ‘joined up’ working across care and custody. Short et al.^30^ found that prison staff struggled with their dual custodial and welfare role while supporting prisoners who had harmed themselves. They lacked training and skills in the welfare elements of their jobs. Similarly, Turner et al.'s^29^ examination of palliative care in prison concluded that the philosophies of care versus custody were problematic. They called for open debates, clear policy and training to ensure that both custodial and care needs were respected and adequately met. Our findings add further support for this notion and suggest a need for clear memorandums of association for integrated health, social and custodial teams.

Health and social care and custodial silos have been inadvertently created in an attempt to meet the dual requirements of care and custody, resulting in a lack of meaningful and effective collaborative working. The existence of silos appeared to have impacted negatively on the successful implementation of the OHSCAP. Indeed, organisational silos and inter-profession tensions have created barriers to improving health outcomes across the public sector and beyond.^31^ The care versus custody dichotomy in prison establishments, further impedes effective multi-agency working. Previous research has found additional evidence of a ‘silo mentality’ in prisons.^32,33^ Bechelli et al.^33^ emphasised the increased costs that are incurred when health and custodial organisations do not work effectively together. This particular study was conducted in the USA, but these increased costs likely apply globally. Moore and Hamilton^32^ found that a ‘silo mentality’ adversely affected the successful operation of resettlement pathways in the UK. They described dysfunctionality within prisons and how organisational silos resulted in a lack of effective communication and shared knowledge. They highlighted a need for further research to enhance understanding of these issues and produce evidence that can be applied in addressing ‘silo mentalities’ in prison that negatively impact upon both staff and prisoners. Empirical evidence relating to interventions for breaking down these silos in prison is lacking.

The OHSCAP facilitators’ efforts to meet health and social care needs were often process-focussed, with a lack of attention to whether or not the desired outcomes were achieved. Rigidity appeared to be a survival tactic for prison officers attempting to cope in challenging, emotive and, at times, violent scenarios and environments. This is comparable to Crawley et al.'s^34^ findings that prison officers frequently prefer not to express emotion or empathy as part of their ‘performance attitude’. The origins of this attitude requires further exploration to ensure that older prisoners’ health and social care needs can be met, and that staff can be appropriately supported.

### Strengths and limitations

A potential limitation of our study is that it took place when the prison service in England and Wales was undergoing a radical transformation, including the Introduction of the Care Act as well as a marked reduction in prison officer numbers.^35^ However, it is important to evaluate such initiatives in ‘real life’ settings given that public services will often experience profound change.^36^ The problems identified were largely systemic, cultural issues that needed to change. The themes were recurrent and they concur with previously published research findings.^32–34^

### Implications for practice

There are important public health implications for ensuring that older prisoners’ health and social care needs are adequately met. There is a duty to ensure that older prisoners receive healthcare services that are equivalent to those received by their peer who reside in the community.^18^ Older prisoners should not be ‘doubly punished’ by loosing their freedom as well as access to equivalent health and social care services.^37^

Furthermore, when prisoners’ health and social care needs are adequately met, although, the direct cost to public services can be high, the resulting gains may offset the initial expenditure.^38^ For example, meeting individuals’ health and social care needs while they are incarcerated may help to reduce reoffending rates post-release, and to thereby contribute towards bringing down the size of the prison population in the longer term.^38^ Also, unmet health needs among older prisoners are associated with depressive symptoms.^39^ Consequently, prison represents an important opportunity to engage with socially disadvantaged and vulnerable groups, such as prisoners to prevent ongoing health and social care needs and associated risks for a range of adverse outcomes, including reoffending, hospitalisation, suicide and other causes of premature death, etc. on release.^40^

It may be possible for the OHSCAP to better meet the health and social care needs of older prisoners if a number of factors are addressed. Facilitators of the OHSCAP should have knowledge and experience of, and interest in, older prisoners. This, accompanied by clarification of the nature of their role, would assist in establishing how to care for older adults sensitively and humanely within a custodial setting.

Interventions are needed to foster more meaningful and effective partnership working and information sharing between prison and healthcare staff. Such initiatives include joint training, designated ‘information sharing and collaboration leads’ within each relevant organisation and the development of clear policies to assist staff in understanding what kinds of information can and cannot be appropriately shared.

Measures can be established to minimise the effects of silos and divisive cultures, such as, information sharing policies, shared training and multi-disciplinary meetings. However, health and social care initiatives, such as the OHSCAP, are likely to have limited success in prisons in England and Wales within the current culture of organisational silos; process-focused solutions, and confusion over how to balance custodial versus health and social care needs. Cultural and strategic change is required for interventions, such as the OHSCAP, to be successfully implemented into prison settings.

The concept that organisational culture can play a significant role in promoting more integrated system approaches is growing across health and social care systems.^41^ Cultural change is difficult but possible in prison. There have been some attempts to use cultural change as a mechanism to improve outcomes in prison, such as reduced risk suicide and non-fatal self-harm. This included ensuring that suicide risk was a central consideration in strategic decision making, and that high-quality listening skills and compassion were encouraged and practised.^42^ It is through such cultural change that the most radical changes to the health and social care of prisoners can be made.

Alternatives to custody should also be considered for older adults with complex health and social care needs. These include release on temporary licence, secure nursing homes, specialised care facilities and compassionate release.

### Future research

A full training-needs assessment of the knowledge and skills of prison and healthcare staff concerning older prisoner issues should be completed. Prisons are unique, and discrete, environments that differ significantly from either home or other institutional settings in which older people are cared for. Therefore, focused ethnographic investigations should be conducted to yield a better understanding of the ways by which the prison environment, prison staff and younger/age-matched peers interact with and affect/influence the day-to-day lives of older prisoners, particularly with regard to meeting their health and social care needs.

An examination of multi-agency and multi-disciplinary working at a more strategic level would be most beneficial in breaking down these barriers. Most importantly, a detailed exploration of the fundamental cultural changes that are required, including how to effectively make these happen, is needed to thereby create a compassionate and integrated custodial, health and social care workforce.
